# Chronic Pain in Patients with Spinal Muscular Atrophy in Switzerland: A Query to the Spinal Muscular Atrophy Registry

**DOI:** 10.3390/jcm13102798

**Published:** 2024-05-09

**Authors:** Leonie Steiner, Anne Tscherter, Bettina Henzi, Mattia Branca, Stefano Carda, Cornelia Enzmann, Joël Fluss, David Jacquier, Christoph Neuwirth, Paolo Ripellino, Olivier Scheidegger, Regina Schlaeger, Bettina Schreiner, Georg M. Stettner, Andrea Klein

**Affiliations:** 1 Division of Neuropediatrics, Development and Rehabilitation, Department of Pediatrics, Inselspital, Bern University Hospital, University of Bern, 3012 Bern, Switzerland; 2 Institute of Social and Preventive Medicine, University of Bern, 3010 Bern, Switzerland; 3 Department of Clinical Research, University of Bern, 3010 Bern, Switzerland; 4 Department of Clinical Neuroscience, Lausanne University Hospital (CHUV), 1005 Lausanne, Switzerland; 5 Division of Neuropediatrics and Developmental Medicine, University Children’s Hospital Basel (UKBB), University of Basel, 4001 Basel, Switzerland; 6 Neuropediatric Unit, Children’s Hospital, University Hospital of Geneva, 1205 Geneva, Switzerland; 7 Pediatric Neurology and Neurorehabilitation Unit, Lausanne University Hospital, 1005 Lausanne, Switzerland; 8 Neuromuscular Diseases Unit/ALS Clinic, Cantonal Hospital St. Gallen, 9000 St. Gallen, Switzerland; 9 Department of Neurology, Neurocenter of Southern Switzerland EOC, 6900 Lugano, Switzerland; 10 Faculty of Biomedical Sciences, Università della Svizzera Italiana, 6900 Lugano, Switzerland; 11 Centre for Neuromuscular Diseases, Department of Neurology, Inselspital, Bern University Hospital, University of Bern, 3010 Bern, Switzerland; 12 Neurology Clinic and Policlinic, Departments of Head, Spine and Neuromedicine, Clinical Research and Biomedical Engineering, University Hospital Basel, University of Basel, 4001 Basel, Switzerland; 13 Department of Neurology, University Hospital Zurich, 8032 Zurich, Switzerland; 14 Neuromuscular Center Zurich, Department of Pediatric Neurology, University Children’s Hospital Zurich, University of Zurich, 8032 Zurich, Switzerland

**Keywords:** spinal muscular atrophy, chronic pain, patient-reported outcome measure, Swiss-Reg-NMD

## Abstract

**Background and Objectives:** Chronic pain is a common symptom in various types of neuromuscular disorders. However, for patients with spinal muscular atrophy (SMA), the literature regarding chronic pain is scarce. Thus, this study assessed the prevalence of chronic pain in children, adolescents, and adults with SMA and investigated the influence of clinical characteristics on chronic pain. **Materials and Methods:** This study used data from 141 patients, which were collected by the Swiss Registry for Neuromuscular Disorders. Extracted data included information on pain (present yes/no, pain location, and pain medication) and clinical characteristics, such as SMA type, motor function, wheelchair use, scoliosis, and contractures. **Results:** The analyses revealed that the highest prevalence of chronic pain was observed in adolescents with 62%, followed by adults with 48%, children (6–12 years) with 39%, and children < 6 years with 10%. The legs, back, and hips were most frequently reported as pain locations. Sex (females), age (adolescents), and the presence of contractures and scoliosis (with surgery) were factors that were associated with chronic pain. **Conclusions:** These findings contribute to a better understanding of pain in SMA, shedding light on its prevalence and characteristics in different age groups, which underscores the importance of assessing and managing pain in patients with SMA.

## 1. Introduction

Over the last decade, an increasing number of studies have highlighted that chronic pain is a common symptom in various types of neuromuscular disorders (NMDs) [1,2]. The causes of pain in neuromuscular disorders are likely multifaceted. They can stem from severe contractures, osteoporosis, vertebral fractures, hip dislocation, orthopedic procedures, or muscular fatigue—all of which are secondary consequences of significantly reduced muscle strength and limited mobility [2,3,4]. Studies in adults with NMDs have reported pain prevalence exceeding 60% [5,6,7], whereas for patients with spinal muscular atrophy (SMA) specifically, the literature regarding pain prevalence is scarce.

Chromosome-5q-associated SMA is an autosomal recessive, degenerative neuromuscular disorder characterized by loss of alpha motor neurons in the spinal cord and brainstem. It leads to progressive muscle weakness and respiratory and bulbar impairment [8]. SMA was historically classified into subtypes 0 to 4 depending on age at manifestation and maximum motor functions achieved [9,10].

Only three studies so far have investigated pain in patients with SMA where children and adolescents were part of the study population. It was shown that the prevalence of chronic pain in type 2 (mean age 17.3 ± 11.7 years) and type 3 SMA patients (mean age 44.9 ± 21.6 years) was 40.6% and 40.9%, respectively [11]. Pain intensity in SMA patients was mild, but pain usually occurred daily, for prolonged durations, and most often in the neck, back, and lower extremities. Sitting and high physical activity exacerbated pain the most [11].

Recently, a study showed that the prevalence of pain for adolescents with SMA was even higher than for adults, with 69% of adolescents experiencing pain in the preceding three months and 50% reporting having chronic pain [12]. Interestingly, the prevalence of pain did not differ between diagnostic groups of patients with neuromuscular disorders (SMA, Duchenne, and Becker muscular dystrophy) nor between those who could ambulate and those who could not. Common pain-exacerbating factors were sitting, too much movement, and being lifted or transferred. Daily activities and participation were negatively influenced by pain [12].

Pitarch-Castellano, et al. [13] showed that the prevalence of pain in a group of children and adolescents with SMA was 43% with a median duration of pain of 5 years. A positive correlation between higher functional levels and frequent lower limb contractures with elevated pain levels was reported. However, they did not examine children and adolescents separately, which is important as prevalence might not be the same.

The aim of this study was to investigate the prevalence of chronic pain in children, adolescents, and adults with SMA in Switzerland and to investigate the influence of clinical characteristics on chronic pain.

## 2. Materials and Methods

### 2.1. Study Design

This study used data collected by the Swiss Registry for Neuromuscular Disorders (Swiss-Reg-NMD), which is approved by the Cantonal Ethics Committee of Bern (20 June 2018, KEK Bern, 2018-00289). The registry was created in 2008 and has been hosted since 2017 at the Institute of Social and Preventive Medicine (ISPM) in Bern. It prospectively collects data of patients of all ages diagnosed with SMA, dystrophinopathies, LAMA2-related muscular dystrophy, or Collagen-6-related muscle diseases in Switzerland. Patients are identified in regional neuromuscular centers. After consent is obtained, the treating physicians report the patient’s baseline data to the Swiss-Reg-NMD and regularly provide follow-up data on the clinical status and treatment of the patient. Initially, this was performed via semi-structured reports; since 2018, a pre-defined case report form (CRF) has been used.

### 2.2. Data Collection

In the CRF, a broad range of data during routine care visits is collected. For this study, we extracted data from the registry including SMA type, motor function, wheelchair use, scoliosis, scoliosis surgery, contractures, and Clinical Global Impression—severity scale (CGI-S). The CGI-S is a 7-point scale that requires the clinician to rate the severity of the patient’s illness at the time of assessment. Possible ratings are normal, not at all ill, borderline ill, mildly ill, moderately ill, markedly ill, severely ill, and among the most extremely ill patients. In each follow-up visit, patients were also asked if they experienced chronic pain that lasted at least 3 months since the last visit. Furthermore, the location and the intake of pain medication were investigated using multiple choice questions within the CRF. Additionally, it was recorded in the registry if patients had physiotherapy. However, it was not recorded if physiotherapy focused on pain. All patients in the registry had physiotherapy, which was therefore not further analyzed, as no detailed information was available on the physiotherapy sessions. Other possible treatments to reduce pain were not recorded in the registry.

### 2.3. Study Population

This study used data from patients collected until 31 December 2023. All patients with a confirmed SMA diagnosis and available data on pain were included in the study. Exclusion criteria were death and missing information on pain.

The introduction of disease-modifying treatments in Switzerland in 2017 has changed the trajectories of patients with SMA tremendously. Today, the Swiss SMA cohort consists on the one hand of young patients treated early and on the other hand of a large number of patients for whom disease-modifying treatments were started late in the course of their disease or not at all. Therefore, the population was analyzed once with all patients and once with only patients over 6 years old.

### 2.4. Statistical Analysis

For the statistical analysis, the Statistical Package for the Social Sciences (SPSS), version 21, and Stata 17.0 were used. Descriptive statistics, with numbers and percentages of demographic and clinical characteristics, prevalence, and frequency, were used. To compare clinical characteristics between groups (pain and no pain), Fisher’s exact test was used to compare the categorical and binary variables.

Multivariable logistic regression was conducted to investigate the influence of clinical characteristics of patients on chronic pain. For the pain group, clinical characteristics were extracted at pain onset. For the no pain group, clinical characteristics were extracted at the last follow-up visit with available information. Regressors were sex, age, SMA type (1, 2, 3), mobility (non-sitters, sitters, walkers), wheelchair use (no use, part-time, full-time), contractures (no/yes), and scoliosis (no, yes w/o surgery, yes with surgery). The total number of patients selected for the regression model was 105 (patients under 6 years old were excluded). The total number of patients used in the multivariate model (without missing values) was 103.

## 3. Results

### 3.1. Study Population

Up until 31 December 2023, 162 patients with SMA were included in the Swiss-Reg-NMD (not reported as deceased; see Table S1 for demographics). Of the 162 patients, 141 (86.5%) patients had available data regarding chronic pain (>3 month), including information on pain location. The main analysis of this study focused on the 141 patients with available pain data from 987 follow-up visits. The study population had an age range between 1 and 66 years, with a median age of 18 years, and 43% were female. Concerning SMA type, there were 28 (19.9%) patients with type I, 59 (41.8%) patients with type 2, 51 (36.2%) patients with type 3, and 3 patients (2.1%) unclassified, as they were treated while asymptomatic. Scoliosis was present in 75.8% of patients, and contractures were present in 63.8% of patients. Concerning mobility, 19.2% patients were walkers, 50.4% sitters, and 30.5% non-sitters. The demographics of the study population were comparable to the entire registered SMA population in Switzerland (Table S1).

### 3.2. Prevalence of Chronic Pain

**Figure 1:**
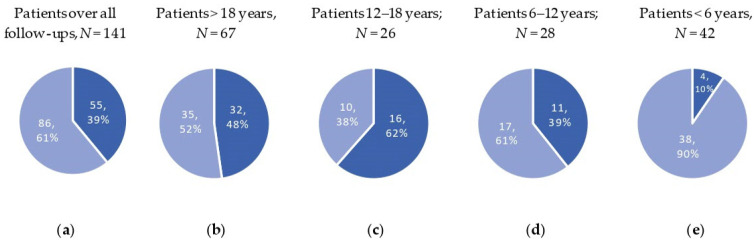
Prevalence of chronic pain overall and in four age groups. Dark blue = chronic pain reported, light blue = no pain reported. Description of reported pain counts (*n*) and percent (%) for (**a**) all patients and all follow-up visits; (**b**) pain group in adults; (**c**) pain group in adolescents; (**d**) pain group in children between 6 and 11 years; (**e**) pain group in children under 6 years. Note that individual patients can appear in more than one age group, as they were followed over time.

The prevalence of chronic pain was first calculated for all patients with available pain data (Figure 1a). In total, 55 patients (39%) reported having experienced chronic pain in one (or more) of the follow-up visits in clinical care, whereas 86 (61%) of the patients never reported having experienced chronic pain up until 31 December 2023.

Secondly, the prevalence was calculated for different age groups separately, which is presented in Figure 1b–e. In total, 48% of the adults, 62% of the adolescents (12–18 years), 39% of the children between 6 and 11 years, and 10% percent of the children under 6 years reported having experienced chronic pain.

### 3.3. Pain Locations

**Figure 2:**
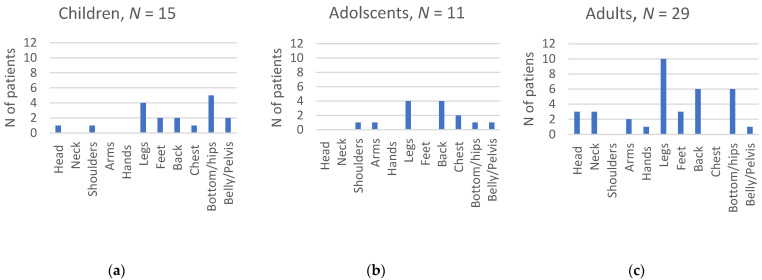
Different pain locations are listed for (**a**) 15 children under 12 years of age; (**b**) 11 adolescents; and (**c**) 29 adults. Information on pain location was extracted at the start of pain. If patients reported pain in more than one location, each location was counted separately.

In the pain group, the most frequently reported pain sites were the legs, back, and hips (Figure 2), with 21% reporting pain in more than one site. Head and neck pain was mostly reported by adults, whereas chest pain was only reported by children (<12 years) and adolescents (Figure 2). The four children under 6 years reported pain in their head, legs, feet, hips, and belly/pelvis.

### 3.4. Clinical Characteristics of Different Age Groups with Chronic Pain

For a detailed overview of clinical characteristics, such as type of SMA, ambulation, scoliosis, scoliosis surgery, and contractures, see Table 1. Clinical characteristics are presented for different age groups at the time when they first reported having chronic pain. Twenty-six patients with chronic pain underwent scoliosis surgery. Of those, only two experienced chronic pain before surgery. Six reported chronic pain 1–6 months after surgery, and the other eighteen patients reported chronic pain 1–4 years after scoliosis surgery.

Pain medication was taken by 28 patients in at least one of the follow-up visits when they reported having experienced chronic pain. Interestingly, only 44.8% of adults took pain medication, compared to 81.8% of adolescents. Furthermore, in children under 6 years (mean age = 4.13 years), three out of four (75%) were taking pain medication.

### 3.5. Clinical Characteristics of Patients Compared between Pain Groups

For group comparisons, patients under 6 years were excluded, as only four patients reported having experienced chronic pain and they reflect a different population of early treated patients. Clinical characteristics, including information on scoliosis, contractures, wheelchair use, and motor function of patients with chronic pain versus patients without pain, are listed in Table 2. In particular, female sex and the presence of contractures and scoliosis (with surgery) are factors that were associated with chronic pain (Table 2). Adolescents and patients with SMA type 2 had a greater odds of pain (OR 2.40 and 2.94, respectively), albeit with large confidence intervals.

### 3.6. Multivariable Logistic Regression Model

Results of the multivariable logistic regression are presented in Table 3. They revealed that sex, age, mobility, contractures, and scoliosis had a potential association with the presence of pain. There was a strong increase in pain in the presence of scoliosis, particularly for those with scoliosis and surgery (OR 10.1, 95%CI [1.53, 11.0]). Females had a greater odds of having chronic pain in comparison to males (OR 3.27, 95%CI [1.14, 9.37]). Additionally, there was an increase of pain in the adolescent group (OR 3.61, 95%CI [0.76, 17.1]). Similarly, there was a potential increase in pain in sitters (OR 2.12, 95%CI [0.26, 17.5]) and non-sitters (OR 2.67, 95%CI [0.24, 29.3] compared to walkers, as well as in the presence of contractures (OR 2.03, 95%CI [0.54, 7.65]), which was not significant because of the large confidence intervals.

## 4. Discussion

The aim of this study was to investigate the prevalence of chronic pain in children, adolescents, and adults with SMA in Switzerland and to investigate its association with clinical characteristics. The prevalence of chronic pain (>3 months) in patients with SMA differed according to age. The highest prevalence was observed for adolescents, with 62%, followed by adults (48%), children (6–12 years; 32%), and children under 6 years of age (9%). The legs, back, and hips were most frequently reported as pain locations. Sex (females), age (adolescents), and the presence of contractures and scoliosis (with surgery) were factors that were associated with chronic pain.

### 4.1. Prevalence in Different Age Groups

The high prevalence rate observed among adolescents aligns with earlier research [12,13]. Lager and Kroksmark [12] emphasized the frequent occurrence of pain in adolescents with SMA (69%). The prevalence rate of pain in children and adults was unclear until now, as there were mixed results. Previous findings indicated that 49% of children with SMA type 2 experienced hip pain [14], whereas in another study, it was found to be 58% in a broader SMA population surveyed via telephone [5]. Uchio et al. [11] identified chronic pain in 40% of SMA type 2 and 3 patients within a combined adult and adolescent population. In a recent study focusing on ambulant adult SMA type 3 patients, it was reported that 55% of the population experienced pain [15]. The variability in reported prevalence rates may stem from the use of distinct methodologies in evaluating pain experiences and the absence of a standardized definition for chronic pain within the context of SMA. The divergence in assessment criteria underscores the need for a more uniform and precise approach to pain measurement in research studies and clinics, thus ensuring a clearer understanding of pain prevalence across different populations and age groups within the SMA population.

Concerning interventions to reduce pain, 28 patients took pain medication in one of the follow-up visits where they reported chronic pain. Interestingly, administration of pain medication differed greatly between groups, with more adolescents (81.8%) and children (75%) taking pain medication than adults (44.8%). The low rate of pain medication intake in adult patients might reflect the moderate or mild pain in this age group, or the development of other strategies to cope with the pain.

### 4.2. Association of Different Clinical Characteristics with Pain

The comparisons between the pain and no pain groups and the regression analysis revealed several differences. There were increased odds of experiencing pain in the presence of contractures and scoliosis, particularly for those with scoliosis and surgery. In the context of SMA, contractures are a common occurrence and have previously been linked to chronic pain [13]. The most reported cause of pain in SMA includes scoliosis, which is consistent with our study findings [12]. Regarding mobility, it was reported that sitting exacerbated pain the most in a questionnaire study [11], which is in line with elevated pain for non-ambulatory patients in our findings. Others found no correlation between ambulatory status and pain [5,12]. Pitarch-Castellano et al. [13] reported a positive correlation between higher functional levels and elevated pain levels, which is contrary to our findings. This contradiction might be due to a different study population and/or a different statistical model used to analyze the association. They analyzed the influence of different clinical characteristics for each variable alone, whereas we included the intercorrelations between various clinical factors in the regression model.

Females were found to have higher odds of having chronic pain than males, which has not been reported before in the literature on the SMA population. However, as the sample size was relatively small and the confidence interval derived from the model was large, the interpretation should be interpreted as exploratory rather than confirmatory.

What remains unclear is how pain affects daily living in our population, as this was not measured. Activities and participation were negatively associated with pain previously [12]. Pain often persists even after orthopedic corrections and can be exacerbated by activities, such as transferring from a bed to a wheelchair [12]. Additionally, in patients with cerebral palsy, pain significantly affected sitting for extended periods, followed by sleeping and transfers [16]. The low muscle mass relative to body weight in SMA results in earlier fatigue, reduced muscle shock absorption, and increased joint loading. These factors contribute to lower cartilage volumes, leading to arthritis, which can cause pain and functional loss [5]. It is essential to consider these complex interactions within the broader context of SMA [5].

Managing pain in patients with SMA begins with the recognition that there are predictable etiologies leading to pain in SMA and investigating them during each medical visit. 

### 4.3. Limitations

Our study has limitations. The case report form is filled out by the clinician and is not directly reported by the patient. Pain might therefore be underreported. The case report form also lacks details about pain and its impact on daily life. We recognize that we do not know the effect of some variables that were not included, such as different treatments to reduce pain (e.g., physiotherapy focusing on pain), psychological state, the effect of concomitant medication, participation in activities, etc. Additional information would add context and deepen the interpretation of our results.

## 5. Conclusions

These findings contribute to a better understanding of pain in SMA patients, shedding light on its prevalence and characteristics in different age groups. The study underscores the importance of assessing and managing pain in SMA patients, as it appears to be an important aspect of their overall health and well-being. Pain is an important factor for quality of life and has an impact on participation [14], which further influences quality of life and needs more attention. Awareness should be heightened among health care providers that pain can be an important issue in patients with SMA.

## Figures and Tables

**Table 1:** Characteristics of patients with chronic pain at the time point of pain onset, shown for pain group overall and for different age groups.

Variables	Pain Yes (All) N = 55	Adults > 18 N = 29	Adolescents 12–18 N = 11	Children 6–12 N = 11	Children 0–6 N = 4
Sex					
Male	27 (49%)	15 (52%)	5 (46%)	5 (46%)	2 (50%)
Female	28 (51%)	14 (48%)	6 (54%)	6 (54%)	2 (50%)
Age group					
Mean (sd)	24.23 (15.50)	35.85 years (11.33)	15.33 years (1.74)	9.79 years (2.03)	4.13 years (1.97)
SMA type					
Type 1	4 (7.3%)	0 (0%)	1 (9.1%)	2 (18.2%)	1 (25.0%)
Type 2	33 (60%)	16 (55.2%)	6 (54.5%)	8 (72.7%)	3 (75.0%)
Type 3	18 (32.7%)	13 (44.8%)	4 (36.4%)	1 (9.1%)	0 (%)
Mobility					
Non-sitter	20 (36.4%)	15 (51.7%)	2 (18.2%)	3 (27.3%)	0 (%)
Sitter	27 (49.1%)	11 (37.9%)	5 (45.5%)	7 (63.6%)	4 (100%)
Walker	8 (14.5%)	3 (10.3%)	4 (36.4%)	1 (9.1%)	0 (%)
Wheelchair use					
Full-time	42 (76.4%)	23 (79.3%)	7 (63.6%)	10 (90.9%)	2 (50.0%)
Part-time	5 (9.1%)	3 (10.3%)	1 (9.1%)	0 (%)	1 (25.0%)
No	7 (12.7%)	3 (10.3%)	3 (27.3%)	1 (9.1%)	0 (%)
Unknown	1 (1.8%)	0 (0%)	0 (0%)	0 (0%)	1
Contractures					
Yes	41 (74.6%)	20 (69%)	9 (81.8%)	9 (81.8%)	3 (75%)
No	13 (23.6%)	8 (27.6%)	2 (18.2%)	2 (18.2%)	1 (25%)
Unknown	1 (1.8%)	1 (3.4%)	0 (0%)	0 (0%)	0 (%)
Scoliosis					
Yes	45 (81.8%)	24 (82.8%)	9 (81.8%)	10 (90.9%)	2 (50%)
No	10 (18.2%)	5 (17.2%)	2 (18.2%)	1 (9.1%)	2 (50%)
Scoliosis surgery					
Yes	26 (57.8%)	14 (75%)	3 (33.3%)	9 (90%)	0 (0%)
No	18 (40%)	9 (45%)	6 (66.7%)	1 (10%)	2 (100%)
Unknown	1 (2.2%)	1 (5%)	0 (0%)	0 (0%)	0 (0%)
CGI-S score (n = 31)					
Borderline affected	1 (3.2%)	-	1 (12.5%)	-	-
Mildly/moderately affected	9 (29.0%)	5 (33.3%)	2 (25%)	1 (20%)	1 (33.3%)
Markedly/severely affected	21 (67.7%)	10 (66.7%)	5 (62.5%)	4 (80%)	2 (66.7%)

Note: data presented as n (%) unless otherwise noted.

**Table 2:** Characteristics of patients with chronic pain versus patients without pain with information on scoliosis, contractures, wheelchair use, and motor function.

	Total	No Pain	Pain	OR (95%-CI)
	N = 105	N = 54	N = 51	
Sex				
Male	62	37 (60%)	25 (40%)	ref. group
Female	43	17 (40%)	26 (60%)	2.26 (1.02 to 5.01)
Age in groups (years)				
Children 6–11	23	12 (52%)	11 (48%)	ref. group
Adolescents 12–18	16	5 (31%)	11 (69%)	2.40 (0.63 to 9.14)
Adults > 18	66	37 (56%)	29 (44%)	0.86 (0.33 to 2.11)
SMA type				
Type 1	8	5 (63%)	3 (38%)	ref. group
Type 2	47	17 (36%)	30 (64%)	2.94 (0.62 to 13.86)
Type 3	50	32 (64%)	18 (36%)	0.94 (0.20 to 4.39)
Mobility				
Walker	20	12 (60%)	8 (40%)	ref. group
Sitter	50	27 (54%)	23 (46%)	1.28 (0.45 to 3.66)
Non-sitter	35	15 (43%)	20 (57%)	2.00 (0.65 to 6.11)
Wheelchair use				
No use	17	10 (59%)	7 (41%)	ref. group
Part-time	10	6 (60%)	4 (40%)	0.95 (0.19 to 4.68)
Full-time	77	37 (48%)	40 (52%)	1.54 (0.53 to 4.48)
Missing	1	1 (100%)	0 (0%)	
Contractures				
No	35	23 (66%)	12 (34%)	ref. group
Yes	69	31 (45%)	38 (55%)	2.35 (1.01 to 5.46)
Missing	1	0 (0%)	1 (100%)	
Scoliosis in categories				
No scoliosis	24	16 (67%)	8 (33%)	ref. group
Scoliosis w/o surgery	45	28 (62%)	17 (38%)	1.21 (0.43 to 3.44)
Scoliosis with surgery	36	10 (28%)	26 (72%)	5.20 (1.70 to 15.92)

Notes: CI, confidence interval; w/o, without; ref., reference. Clinical characteristics were extracted at pain onset. For the no pain group, clinical characteristics were extracted at the last follow-up visit with available information.

**Table 3:** Results of the multivariable logistic regression. Complete cases analysis (N = 103).

Presence of Pain/Variables	OR (95%-CI)	*p*-Value
Sex (ref. male)	3.27 (1.14 to 9.37)	0.028
Age (ref. 6–12 years)		
Adolescents 12–18	3.61 (0.76 to 17.1)	0.105
Adults > 18	1.65 (0.43 to 6.30)	0.460
SMA type (ref. type 1)		
Type 2	1.32 (0.22 to 7.82)	0.763
Type 3	0.77 (0.08 to 7.14)	0.818
Mobility (ref. walker)		
Sitter	2.12 (0.26 to 17.5)	0.484
Non-sitter	2.67 (0.24 to 29.3)	0.422
Wheelchair use (ref. no use)		
Part-time	0.58 (0.09 to 3.65)	0.565
Full-time	0.11 (0.01 to 1.22)	0.073
Contractures (ref. no)	2.03 (0.54 to 7.65)	0.297
Scoliosis (ref. no)		
Yes w/o surgery	1.64 (0.33 to 8.10)	0.543
Yes with surgery	10.1 (1.53 to 11.0)	0.016

Note: OR, odds ratio; CI, confidence interval; w/o, without; ref., reference.

## Data Availability

All data included in this study are recorded in the Swiss-Reg-NMD registry. Data can be obtained anonymized and aggregated upon reasonable request to the corresponding author (L.S. or A.K.) and after approval of the registry’s steering board.

## Supplementary Materials

The following supporting information can be downloaded at: https://www.mdpi.com/article/10.3390/jcm13102798/s1, Table S1: Demographics of Swiss SMA population. Table S2: Swiss-Reg-NMD Group.
