# Supplementary material for: Chronic Pain in Patients with Spinal Muscular Atrophy in Switzerland: A Query to the Spinal Muscular Atrophy Registry
Source: J Clin Med. 2024 May 9;13(10):2798. doi: 10.3390/jcm13102798 (PMC11122245; doi:10.3390/jcm13102798)
Supplement: Supplementary file 1 [file jcm-13-02798-s001.zip › jcm-2963079-supplementary.pdf]

Supplementary Materials

The demographics of the Swiss SMA population (N=162) and the included study group (N=141) are described in table S1. Patients age ranged between 1 - 66 years with a median age of 21 years and 43% were female. Concerning SMA type, there were 29 (17.9%) patients with SMA type 1, 67 (41.4%) patients with SMA type 2, 62 (38.9%) patients with SMA type 3, and three patients (1.9%) that could not be classified with this nomenclature, as they were treated while asymptomatic. Scoliosis was present in 74.1% of patients, and contractures were present in 63.0% of the patients. Concerning mobility, 20.4% patients were walkers, 50.6% sitters, and 29.0% non-sitters.

Table S1. Demographics of Swiss SMA patients

Variable	Swiss-Reg-NMD patients with SMA (N = 162)	SMA patients included in study (N = 141)
**n/N**	-	86.50%
**age range**		
min	1	1
max	66	66
**age median**	21	18
**sex**		
female	70 (43%)	61 (43%)
male	92 (57%)	80 (57%)
**SMA type**		
1	29 (18%)	28 (20%)
2	67 (41%)	59 (42%)
3	62 (39%)	51 (36%)
non-classified	3 (2%)	3 (2%)
**Scoliosis**	120 (74%)	107 (76%)
**Contractures**	102 (63%)	90 (64%)
**Mobility**		
walkers	33 (20.4%)	27 (19.2%)
sitters	82 (50.6%)	71 (50.4%)
non-sitters	47 (29%)	43 (30.5%)

Table S2. Swiss-Reg-NMD Group

Dominique Baumann	Institute of Social and Preventive Medicine, University of Bern
Cornelia Enzmann	Pediatric Neurology, University Children's Hospital Basel
David Jacquier	Pediatric Neurology and Neurorehabilitation, Lausanne University Hospital
Hans H. Jung	Department of Neurology, University Hospital Zurich
Andrea Klein	Department of Neurology, University Children's Hospital Basel and Inselspital Bern
Claudia E. Kuehni	Institute of Social and Preventive Medicine, University of Bern
Andrea Mathis	Institute of Social and Preventive Medicine, University of Bern
Paolo Ripellino	Neurocenter of Southern Switzerland, Lugano
Oliver Scheidegger	Department of Neurology, Centre for Neuromuscular Diseases, Inselspital Bern
Bettina Schreiner	Department of Neurology, University Hospital Zurich
Esther I. Schwarz	Department of Respiratory Medicine, Sleep Disorders Centre and Neuromus-cular Centre, University Hospital of Zurich
Georg M. Stettner	Department of Pediatric Neurology, University Children's Hospital Zurich
Elea Galiart	Department of Pediatric Neurology, University Children's Hospital Zurich
Anne Tscherter	Institute of Social and Preventive Medicine, University of Bern
